# “My Fibro Family!” A qualitative analysis of facebook fibromyalgia support groups’ discussion content

**DOI:** 10.1080/24740527.2022.2078183

**Published:** 2022-06-28

**Authors:** Lyndsay Crump, Diane LaChapelle

**Affiliations:** Department of Psychology, University of New Brunswick, Fredericton, New Brunswick, Canada

**Keywords:** fibromyalgia, Facebook, online support group, peer support, qualitative methods, chronic pain

## Abstract

**Background:**

Fibromyalgia (FM) is a diagnostically controversial syndrome characterized by chronic widespread pain, fatigue, sleep difficulties, cognitive dysfunction, and mental health symptoms. Though online peer support groups (OPSGs) may help persons with FM access support and information, there are concerns that such groups can be harmful.

**Aims and Methods:**

Using a nonparticipatory observational stance, the authors analyzed discussions in three Facebook FM OPSGs (approximately 15,000 members, mostly women) to determine what themes best characterize their discussion content and whether being in a particular group was related to the type of thematic content to which they were exposed.

**Results:**

Two themes were identified that represented explicit reasons group members participated in the OPSG (trying to understand FM and seeking/offering emotional support). Six themes represented underlying reasons members sought informational and emotional support in FM OPSGs (fighting FM, learning to live with FM, struggling with identity, distressing thoughts and feelings, judgment, empowerment-seeking). No salient differences were identified between the thematic content of each group.

**Conclusions:**

The findings suggest that FM OPSGs may provide much needed psychosocial and emotional support regarding important aspects of psychological adjustment to living with FM while also inadvertently encouraging approaches to living with FM that do not align with evidence-based FM management recommendations (e.g., investment in fighting rather than accepting FM). These findings may be useful to patients considering joining an FM OPSG and to health providers helping patients navigate to resources that can address their emotional or psychological support needs.

## RÉSUMÉ

**Contexte:** La fibromyalgie (FM) est un syndrome dont le diagnostic est controversé et qui se caractérise par des douleurs chroniques généralisées, de la fatigue, des troubles du sommeil, un dysfonctionnement cognitif et des symptômes de santé mentale. Bien que les groupes de soutien par les pairs en ligne puissent aider les personnes atteintes de FM à obtenir du soutien et de l’information, certains s’inquiétent du fait que ces groupes peuvent être nuisibles.

**Buts et méthodes:** En utilisant une position d’observation non participative, les auteurs ont analysé les discussions dans trois groupes de soutien par les pairs en ligne FM sur Facebook (environ 15 000 membres, principalement des femmes) pour déterminer quels thémes caractérisent le mieux leur contenu de discussion et si le fait de faire partie d’un groupe particulier était lié au type de contenu thématique auquel ils ont été exposés.

**Résultats:** Deux thémes représentant les raisons explicites pour lesquelles les membres du groupe ont participé au groupe de soutien par les pairs en ligne ont été recensés (essayer de comprendre la fibromyalgie et rechercher/offrir un soutien émotionnel). Six champs thématiques représentaient les raisons sous-jacentes pour lesquelles les membres cherchaient de l’information et du soutien émotionnel dans les groupes de soutien par les pairs en ligne FM (combattre la fibromyalgie, apprendre à vivre avec la fibromyalgie, avoir des difficultés d’identité, les pensées et sentiments pénibles, le jugement, la recherche de l’autonomie). Aucune différence marquante n’a été observée dans le contenu thématique d’un groupe à l’autre.

**Conclusions:** Les résultats indiquent que les groupe de soutien par les pairs en ligne FM peuvent fournir des services psychosociaux indispensables et un soutien émotionnel concernant des aspects importants de l’adaptation psychologique à la vie avec la FM, tout en encourageant par inadvertance des approches de vie avec la FM qui ne correspondent pas aux recommandations de prise en charge de la FM fondées sur des données probantes (par exemple, investir dans la lutte contre la FM plutôt que de l’accepter). Ces résultats peuvent être utiles aux patients qui envisagent de se joindre à un groupe de soutien par les pairs en ligne FM et aux prestataires de soins de santé qui aident les patients à trouver des ressources qui peuvent répondre à leurs besoins de soutien émotionnel ou psychologique.

## Introduction

Fibromyalgia (FM) is a chronic pain condition characterized by a constellation of persistent and unpredictable somatic, mental health, and cognitive symptoms associated with impairments in physical, emotional, occupational, and social functioning.^1^,^2^ Quality of life for persons with FM typically declines due to these symptoms and FM’s impact on self-esteem, interpersonal relationships, and financial well-being.^3,4^ FM’s unknown etiology and invisible nature also make patients more vulnerable to stigmatization, which can exacerbate social and emotional difficulties.^5,6^

Typical treatment for FM focuses on managing symptoms with medication and physiotherapy/exercise; however, these are only mildly to moderately effective in reducing FM symptom severity and improving functioning.^7–9^ Treatment ideally also includes patient education and psychological therapies that can foster improvements in symptom ratings, functioning, and mental well-being.^1,10–13^ Unfortunately, engagement in psychological interventions is limited by access issues such as cost, proximity to a competent provider, mobility, or transportation difficulties.^14^

Online peer support groups (OPSGs) provide an opportunity to offer and receive support and information from similarly affected peers with minimal perceived threat of stigmatization. Thus, they seem an ideal social support platform for those affected by controversial and difficult to treat illnesses like FM.^15^ Social media sites, such as Facebook, have greatly facilitated the accessibility of OPSGs, connecting millions of people with shared interests or issues globally at little to no cost.^15,16^ Individuals can electronically “log on” day or night to discuss their symptoms, illness-related experiences, or support needs from the comfort of their own home, mitigating access barriers that often make support resources inaccessible for persons with FM.^15,16^ Facebook-based OPSGs may be especially popular among persons with FM because time, geographic, architectural, systemic, and technological barriers (including user accessibility) are circumvented by using Facebook’s online communities.^15,16^

Facebook OPSGs may also be uniquely appealing because they are exceptionally accessible to the large proportion of adults already using Facebook on a daily basis.^17^ Users can find and join a Facebook group using their existing Facebook profile (or, if preferred, a distinct profile created for OPSG participation) and engage with others using a variety of interaction styles (e.g., posting text, pictures, videos, web links; using one-click “like” or emoji responses) from virtually any internet-enabled device.^18^ Users can receive notifications on their account dashboard and smart devices when others have posted content in the group or responded to their posts, potentially prompting them to revisit the group discussion each time they access their smart device or log on to Facebook.^19^ A Facebook OPSG member may also choose not to disclose their real name or other identifying information in the group or may choose to casually consume but not contribute to the group’s discussion content.^20^ Thus, Facebook OPSGs may help some individuals overcome a multitude of barriers that impede more formal help-seeking.

Despite the proliferation of illness-related OPSGs on Facebook, there is very limited research examining groups’ discussion content and, to the authors’ knowledge, no published research that has examined Facebook groups’ discussion content using nonparticipatory observational methodology (i.e., where group members are unaware of the research and thus unlikely to change their behavior). One published study^21^ examining content posted in a Portuguese-speaking Facebook-based FM support community was identified. In this research, the authors found that the group offered opportunities to “vent” about FM-related suffering and stigmatization, to receive emotional support and social contact from the group, and to benefit from “active contributions from the moderators with affectionate and constructive comments.”^21^^(p6)^ Notably, this Facebook group had a formal coordinator, was monitored by 14 administrators, offered training for group discussion moderators who enforced group rules, provided feedback on the credibility of information shared, and was associated with a national patient association and free information channel. This level of group oversight, management, and organizational affiliation is atypical in the context of peer-led Facebook-based support groups. In addition, the group members were aware of, and in some cases directly engaged in, the study of the group. Thus, it is not possible to know whether group members changed their behavior in response to the known presence of the researchers. It is also not possible to know whether the findings are transferable to unmoderated, peer-led Facebook OPSGs.

Research examining what members discuss within technologically older style (i.e., nonsocial media–based groups) peer-led FM OPSGs may offer some insight but is also limited in terms of both the number of studies and their methodological approaches. Two of these studies^22,23^ indicated that group discussion focused on social connectedness (e.g., “chit chat”), treatment information, and sharing support and personal experiences, and the third study^24^ found evidence of potentially harmful discussion topics (e.g., illness reification, derogation of professional expertise in favor of embodied knowledge). Though these findings provide additional insight into what is discussed in FM OPSGs, they are compromised by methodological limitations. Specifically, van Uden-Kraan and colleagues^23^ and Barker^24^ both analyzed the extracted OPSG content using pre-established coding schemes (i.e., a deductive methodological approach), which significantly shaped and limited the types of themes that were identified. In the third study, Chen^22^ used a computer automated linguistic analysis to identify the most common words and word clusters present in the extracted content. Without contextual or semantic information, however, the interpretability of Chen’s research findings is severely limited.

Recently, Berard and Smith^15^ reported that persons with FM are using Instagram to access information, advice, mentorship, emotional support, and camaraderie, which is perceived by users to be empowering and helpful for managing stress associated with living with FM. By analyzing 50 Instagram posts and 12 sets of user-completed questionnaires, these authors identified four themes in the FM narratives posted (“impact of fibromyalgia,” “pain management,” “faces of fibromyalgia,” and “awareness of education”) and noted that participants described the FM Instagram community as a space that provides acceptance and emotional support.^15(p243)^ These findings generally align with previous FM OPSG research.^22,23^ Although this study provides some valuable insights into how patients with FM are using modern social media–based technologies for support, it is limited by the authors’ decision to analyze only 50 posts when “approximately 200,000 posts on FM [are] uploaded every three to four days” on Instagram.^15(p245)^ Analysis of a larger data set may have allowed for the identification of novel themes or a richer exploration and identification of lower-order themes (e.g., impact of FM on identity) that would enhance the current understanding of what is discussed in FM online support communities. Furthermore, communities on Instagram also develop and function very differently from in-person support groups or OPSGs on Facebook because of Instagram’s interaction parameters and emphasis on sharing images/videos rather than text. Instagram users may “follow” other individual users and comment on that user’s posts or may follow or search and comment on content posted with a specific hashtag (e.g., #fibromyalgia), but there is no function (barring individually tagging or messaging every member) that allows users to post content to a designated “group” within the singular worldwide FM Instagram community. Thus, the format and structure of a Facebook OPSG differs from Instagram hashtag communities or poster–follower relationships in fundamental ways.

Given that very little research has explored FM OPSGs, additional research is needed to determine whether the rise of social media–based OPSGs has affected the type of content discussed among members. It is possible that the integration of social media into people’s daily lives has impacted both the types of users who choose to participate in FM OPSGs and the type of content they discuss. We therefore completed an inductive analysis of discussion content in Facebook FM OPSGs with the goals of identifying (1) the themes that best characterize the discussion content of Facebook FM OPSGs and (2) whether being in a particular Facebook FM OPSG is related to the type of thematic content to which users are exposed.

## Methods

This research used nonparticipatory observation, a metho-dology that allows the researcher to silently observe participants’ behaviors and interactions without their knowledge (or, in the context of online environments, “lurk”). Experts recommend nonparticipatory observational field methods when studying online support groups and patient forums because group members are more likely to monitor or change their behavior when they know they are being observed, presenting a distorted picture of group functioning to researchers observing the online environment.^24,25^

The use of nonparticipatory observational research methods requires careful consideration of ethical issues pertaining to privacy and informed consent. According to Article 10.3 of the Canadian Tri-Council policy addressing observational (i.e., noninteractive) qualitative research, researchers must outline why the persons being observed are assumed to have a limited expectation of privacy. Researchers must then justify why “an exception to the general requirement of [informed] consent” is needed in the context of their study.^26(p145)^ With respect to establishing a limited expectation of privacy, only Facebook FM OPSGs that had at least 500 members, were classified as public forums (not secret) and could be joined by simply requesting membership were considered. Although each groups’ administrators must approve requests to join the groups, there were no conditions or criteria that needed to be met for group membership (i.e., the researcher did not have to provide any information or interact with anyone beyond clicking “join” to access the group). Thus, content posted in these groups was considered accessible to any member of the public who requests to join. Groups whose netiquette statements stipulated the discussion content was private or copyrighted were also excluded from consideration.

The Tri-Council^26^ policy outlines five conditions under which exemptions from the general requirement of consent can be granted. The first two conditions stipulate that the research must not exceed minimal risk of harm to participants and must be unlikely to adversely affect their welfare. Group members’ thoughts, feelings, and behaviors were in no way impacted by the researcher’s silent, observational presence and only the comments posted to the group (not user profiles) were analyzed. All disseminated results only identify the groups generically to protect the identity and welfare of the groups and members.

The third exception condition requires that it be “impossible or impracticable to carry out the research and to address the research questions properly, given the research design, if the prior consent of participants is required.”^26(p36)^ Article 10.3 of the Tri-Council^26^ policy states that consent is not necessary when the researcher has no direct interaction with the individuals being observed (e.g., when nonparticipant observation is used). The fourth exemption condition stipulates that, whenever possible and appropriate, participants will be debriefed and provided with additional pertinent information.^26^ Posting a debriefing message on the group’s wall or contacting the thousands of group members would violate the methodological approach and potentially the anonymity of the groups and membership. Instead, published, open access results of this research will be shared with administrators of any searchable Facebook FM OPSGs, who will be encouraged to share it within the group. Finally, the fifth condition, which stipulates that the research does not involve an intervention of any type, is met because the research did not involve the provision of any service such as assessment or treatment (i.e., the research was purely observational).^26^

In addition to meeting the conditions of the Tri-Council^26^ policy regarding ethical conduct for research involving humans, requests to alter or forgo the general requirement of consent must demonstrate that the research results will be of some benefit to individual participants, subpopulations, or society in general. The authors hope this research may help patients with FM better understand the potential costs and benefits of FM OPSGs and to decide whether they wish to participate in an OPSG. Likewise, the research results may help inform health professionals’ decisions about whether to recommend or caution against FM OPSG usage.

### OPSG Selection Procedures

Once this research was approved by the authors’ university research ethics board, an initial list of Facebook FM OPSGs was generated using the Facebook search function. This initial search yielded almost 100 English-speaking groups. Follow-up efforts within days of the initial search to replicate the list of possible groups consistently yielded a list of the same groups in a different order, suggesting that the initial search list was randomly generated. In compliance with the Tri-Council^26^ policy conditions outlined above and Eysenbach and Till’s^27^ criteria for determining whether a group is a private space in the context of online qualitative research, only publicly searchable Facebook FM OPSGs that could be accessed by clicking “join” or that had more than 500 members were considered for selection. Any group whose netiquette statements stipulated the discussion content was private or copyrighted was excluded. Given the authors’ compliance with both the Tri-Council^26^ and Eysenbach and Till’s^27^ conditions, the level of risk to participants was deemed to be minimal in compliance with Canadian federal research policies^26^ and in line with similar policies abroad.^28^

Groups were excluded from consideration if their title indicated (1) an affiliation to a country outside North America, (2) a religious persuasion, (3) inclusion of persons with illnesses other than FM (e.g., chronic pain), (4) specific goals beyond general support (e.g., weight loss, nutrition), or (5) a specific coping stance (e.g., natural recovery, living positively). Exclusions were justified as an attempt to ensure, within the limitations imposed by the authors’ observational methodology, that persons in the groups self-identified as being impacted by FM specifically and to facilitate comparison of group discussions in FM OPSGs that are not driven by a specific theme or tone (e.g., religion or coping stance). Groups that were inactive (i.e., less than one post per 7 days) were also excluded. The first three groups that met inclusion criteria were chosen from this pool of more than 50 groups and “joined.”

When data collection commenced, all messages posted to each group’s wall during the second week of the month (e.g., Month C) were initially collected. To expedite data collection, the first author then backtracked through each group’s archived wall posts to extract all messages posted during the same 7-day period of the previous 2 months (i.e., the second week of Months B and A). One group was so active that the authors were ultimately unable to access archived content posted to the group’s wall during the earliest designated month of data collection (i.e., were only able to collect data from 2 weeks [14 days] rather than 3 weeks [21 days] for this group). Repetitive attempts using multiple web browsers and multiple laboratory computers were made to access these data; however, at each attempt the browser or computer experienced an error before these data became available. All extracted content was copied and pasted into separate documents.

The data set consisted of over 33,000 kilobytes of data, including 591 original posts (i.e., messages, images, videos, or other content that is not linked to previous posts). Facebook also permits users to comment on content posted by others, and taking these comments into account a total of 1121 unique group members posted or commented on material posted to the group walls during the designated data collection period. The groups were not formally moderated. The group “admins” were described as persons with FM with the authority to remove any members posting content that violated the basic group rules or “netiquette” posted in all the groups (e.g., posting profane, unsupportive, or abusive content). None of the groups were associated with any patient-led FM societies and no organization or person owned the groups.

### OPSG Members

The use of nonparticipatory observational methods prohibited the collection of verifiable demographic information. Nevertheless, across the estimated 15,000 group members, those who chose to disclose a gender/sex identity almost exclusively identified as female or women based on their pronoun use, self-presentation (e.g., profile picture), or the content of their posts (e.g., reference to pregnancy, menstruation, mothering). Less than ten members who posted content in the groups identified themselves as male/men. Group members described varying durations of illness experience (e.g., newly diagnosed to 20 years or more of FM experience). Members who included demographic information in their posts also endorsed a range of relationship statuses (e.g., single, common-law, married, divorced) and were diverse in terms of age, spiritual beliefs, cultural backgrounds, and socioeconomic status. Based on the content of their posts, most group members appeared to reside in North America. Less than ten members specifically posted content related to living abroad (e.g., United Kingdom, Australia, Kenya).

### Data Analysis

All extracted content was analyzed according to Braun and Clarke’s^29^ six steps of inductive thematic analysis using NVivo 12 software^69^. This enabled the first author to produce a data-driven description of the major themes that characterized the entire data set (all three FM OPSGs’ content) as well as the themes that characterized each FM OPSGs’ discussion content for comparative purposes. Once familiarized with the data (via multiple readings; phase 1), the first author systematically coded prominent or noteworthy features in the content of each OPSG, collating data under each code to form preliminary coding hierarchies (phase 2). The first author then organized these codes into preliminary themes (phase 3).

Iterative review of the content and codes subsumed within each theme as well as the themes in relation to one another was completed by both authors to ensure that each theme represented a unique aspect of the data. The thematic maps were subsequently generated by the first author (phase 4) and each theme’s name and definition was refined and concretized by both authors (phase 5). Throughout the coding process, codes and themes were reviewed and discussed with the second author and other members of the research lab to ensure that themes were cogently assigned and operationalized. This was an ongoing process throughout data analysis to ensure the trustworthiness (i.e., credibility, dependability, confirmability, reflexivity) of the findings presented here in phase 6.^30,31^

### Researcher Frame of Reference

Credible qualitative analysis involves acknowledgment of the reflexive lens through which the research was conceptualized, designed, and executed.^32^ Both authors identify as female/women and have personal experience with chronic pain or other chronic illness within the context of the Canadian medical system. In addition, both authors have doctoral training in clinical psychology and share clinical and research interests in working with persons with FM. These personal and professional experiences undoubtedly impact the authors’ understanding of participants’ experiences with FM and data interpretation.

## Results

Analysis of the data from all three groups indicated no noteworthy differences in each group’s thematic map, suggesting that persons with FM were generally exposed to the same type of thematic content regardless of which group they joined. This finding addressed the secondary goal of this study and justified presentation of the themes that best characterize the discussion content of the Facebook FM OPSGs (primary goal of the study) collectively.

### Reasons Group Members Participate in Facebook FM OPSGs (higher-order Themes)

**Figure 1.**
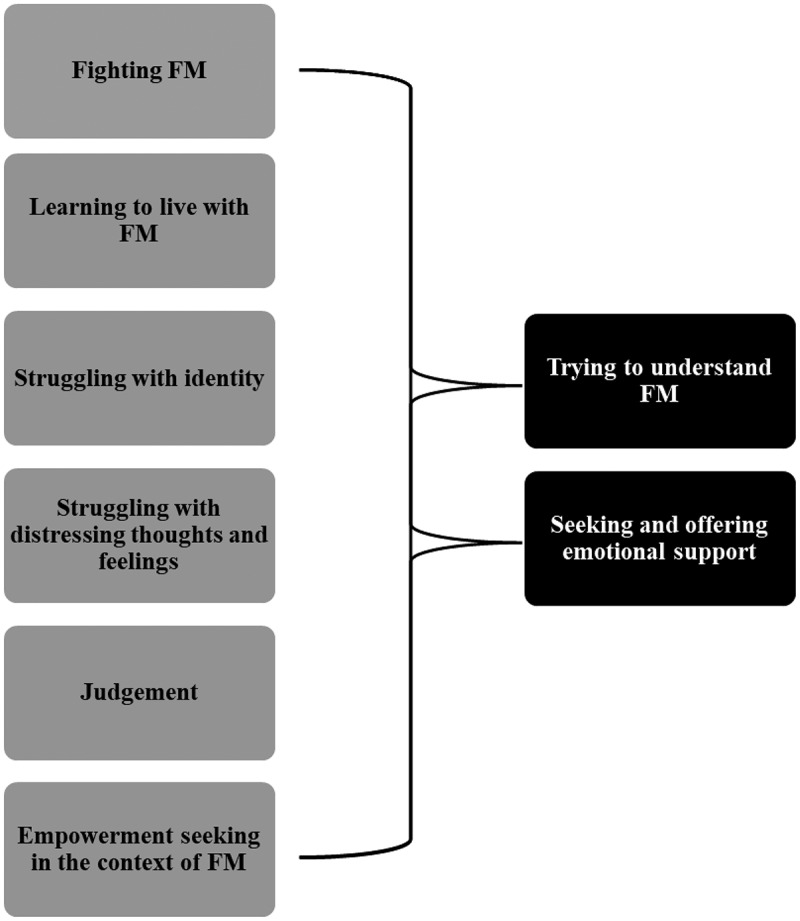
Themes characterizing the discussion content of the Facebook FM OPSGs. Note. Higher-order themes are indicated by black figures and lower-order themes underlying the higher-order themes are indicated by gray figures.

Analysis of all three groups’ posts and their affiliated comments yielded eight themes (see Figure 1). The authors identified two higher-order themes in the content posted to the groups’ walls representing reasons group members participated in a Facebook FM OPSG (trying to understand FM and seeking and offering emotional support). Six lower-order themes representing why members seek information and emotional support online (fighting FM, learning to live with FM, struggling with identity, distressing thoughts and feelings, judgment, empowerment-seeking) were also identified. The higher-order themes are presented below, followed by details about the lower-order themes.

#### Trying to Understand FM

Content in every group reflected members’ struggle to find reliable and accurate information about FM. That is, members expressed a desire to better understand what FM is (and is not), why it develops, how to treat their specific symptoms, and how to navigate life with FM more generally. Content reflective of this theme included posts/comments questioning whether specific symptoms are caused by FM (e.g., skin rashes, inflammation), descriptions of specific symptoms associated with FM (e.g., pain, brain fog), and descriptions of other conditions potentially misdiagnosed as FM (e.g., Lyme disease, multiple sclerosis). Other content reflective of this theme included web links to information about theorized origins of FM or particular symptoms (e.g., article suggesting “depression is an allergic reaction to inflammation”) and information about the developmental course and prognosis of FM.

Some of the information provided about the symptoms associated with FM and recommended treatments was consistent with current scientifically based information (e.g., “I have heard of some people improving … enough to be able to call it a ‘cure.’ But they find they still get flare ups if they do too much”). Occasionally, group members posted information with a less credible scientific basis (e.g., article claiming FM pain stems from excess blood vessels in the hands) or information that would be difficult for consumers without advanced medical knowledge to interpret (e.g., article exploring mitochondrial enhancer replacing Cymbalta in treatment of FM). Group members also occasionally posted advertisements or endorsements for products (e.g., branded nutritional supplements).

In addition, severe symptom presentations (e.g., daily intractable pain, severe mobility limitations) were often characterized as typical of FM, and additional symptoms that may be better explained as medication side effects, concurrent health problems, or non-FM normative experiences were also presented as FM symptoms (e.g., irritable bowel, rashes, anxiety, hormone imbalances, dry mouth). Along these lines, FM was regularly described as debilitating, all-encompassing, and progressive. For example, two different members posted: “… the pain is not comprehendible. I am frightened of what the future holds for me”; “I only got worse. I am now deemed permanently disabled.”

#### Seeking and Offering Emotional Support

Consistent with the nature of most support groups, seeking and offering emotional support (i.e., requests for or expressions of empathy, compassion, acceptance, belonging, or investment in others well-being) was identified as a prominent theme in the content posted in all three FM OPSGs. On occasion, members solicited support directly (e.g., “I feel so guilty. I can’t take care of my family and I don’t want to share with them just how bad this is. Please I need someone that understands”). More often, however, emotional support was offered without prompting when members posted about a difficulty. For example, one group member concluded her response to another member’s lengthy description of a stigmatizing experience by stating: “Don’t stress yourself out. Know you are safe to say what you want here and know we will support and understand you!!”

Additional supportive responses, including hope for reduced/no pain, general or personalized encouraging statements, affirmation statements (e.g., “Be happy. Be strong. Believe we will survive”), and offers of prayers were noted in all groups, though this latter form of support (i.e., prayer) was especially prominent in one group. Some participants also offered support by normalizing, legitimizing, or validating members’ FM experiences or life difficulties (e.g., “I really hope [your family] take your needs more in consideration than theirs”) or by offering a sense of connection or belonging to members (e.g., “gentle hugs my fibro family”; “always an ear here and a virtual hug”).

*Venting*, defined as expressions of strong, negatively valanced thoughts or emotions (e.g., anger, betrayal, helplessness) relative to a situation without a solution focus, was a prominent support-seeking mechanism observed in the content collected from all groups. Venting consistently elicited offers of emotional support from other group members. Members generally “vented” about physical symptoms, medication ineffectiveness or side effects (e.g., “[medications] make me so sleepy where I can’t do anything but yet I can’t sleep. It sucks”; “… the sharp electric pain is the worst I’ve ever felt! will it never end?”), and FM’s impact on quality of life (e.g., “… living like this sucks and I hate it”). Members also vented about feeling misunderstood/stigmatized by others who do not have FM (e.g., “I hear too often that I’m whining …”; “I’m so frustrated … people who don’t have FM will never understand”). On some occasions, posters were also observed to vent about the same issue repetitively in their responses to others posts. For example, one member posted: “I am so thankful my ex took off before I was diagnosed and before things got as bad as they are now. I have enough pain and issues going on, I don’t need to deal with him on top of it!” Within a 2-week period, this member posted similar sentiments on two additional occasions.

Venting posts typically prompted other members to offer unsolicited advice or to vent about their own experience, which sometimes resulted in support being redirected toward a responder rather than the original poster. Often (but not always), members offered emotional support including validation, encouragement, normalization, and connection (e.g., “I am sorry that you have had this happen … it takes someone that actually experiences fibro to truly understand and have patience”; “I commend you for all the pain I know you are enduring you aren’t letting it beat you”).

### Reasons Group Members Seek Information or Support in Facebook FM OPSGs (lower-order Themes)

Collectively, trying to understand FM and seeking and offering emotional support were the intuited reasons members joined and participated in the groups in this data set (e.g., “This site has been so amazing for me since joining recently. Finally, people that feel the same way I do. … I find some comfort in knowing I am not alone or crazy”). Further examination of the content, however, revealed six additional themes representing reasons members may seek support or information from peers online rather than (or as an adjunct to) seeking to meet these needs offline with family, friends, or helping professionals.

#### Fighting FM

Posted content indicates a large proportion of members were heavily invested in seeking and offering ways to “fight” FM. In this data set, fighting FM was defined as efforts to eliminate or reduce symptoms or arrest their progression to regain quality of life. Analysis of members’ posts suggests they fought FM by seeking and offering advice about medication, home remedies, alternative therapies (e.g., acupuncture, aromatherapy), medical devices (e.g., transcutaneous electrical nerve stimulation (TENS)), vitamins, complementary and alternative medicines, and other “treatment” regimens for FM (e.g., gluten-free dietary regimens). Some members also discussed avoiding stressors or activities (e.g., “if I take a walk I pay for it for days”) that they perceived could potentially exacerbate their symptoms. Others expressed reluctance to adapt or pace their activities, viewing completing tasks regardless of FM-related limitations or symptom exacerbation as a form of fighting (e.g., “Definitely overdid it. … Needed to get [task] all done. Unfortunately I’ve been up since 4am in excruciating pain”).

Some members also used the term “fight” in the context of raising awareness or fighting negative stereotypes about persons with FM (e.g., “I fight for my health every day in ways most people don’t understand”; “I’m a warrior”; “#FIGHTING4MYLIFE”). Content referring to members as “fighters” or “warriors” was commonplace in the data set and was described as a way to validate and attribute valued qualities of courage, strength, resilience, and persistence to members struggling to live with FM. Exemplifying this point, members posted: “Fibro is painful but as fighters we shall never give up or give in. Gentle hugs my fellow warriors”; “God Bless all of you for your support and the fight that we live.”

#### Learning to Live with FM

Some posted material focused on adapting to rather than fighting FM. Learning to live with FM encompassed content that conveyed members’ efforts to stop fighting FM-related current or anticipated changes in their lives (e.g., accepting reduced working hours or accommodations rather than struggling to overcome limitations in the workplace) in favor of investing in activities that foster improved quality of life and self-acceptance. Group members contextualized this experience of investing in creating purpose and the capacity for joy in life with statements like: “There’s nothing wrong with hope. I just think we just have to stop chasing it exclusively … and start to adjust to the life we have here and now.”

At a more nuanced level, members posted about cultivating joy in their lives by participating in pleasant activities (e.g., enjoying a hot bath, swimming, singing aloud) and increasing time spent with loved ones:

Poster: In spite of pain, I still keep [my grandchildren] whenever possible … [they’re] just a joy.

Responder 1: Cuties! I have three. They certainly encourage me to do as much as I can!!!

Responder 2: Grandchildren help lessen our focus on pain!

Members also described cultivating joy by adapting their surroundings to be more pleasant (e.g., “I put things I feel happy looking at where I’ll see them often”). Members discussed learning to accept accommodations and to plan and pace their activities to complete meaningful tasks without exacerbating symptoms. For instance, one responder encouraged others to pace their activities in this way: “On the worst days, try to do at least one thing that makes you feel good. On the better days, move as much as you can—swim, walk—even if it’s just a little. The more you don’t move, the less you will.”

Lastly, members posted memes depicting self-acceptance (e.g., “Tell yourself gently: I love you, you did the best you could today, and even if you didn’t accomplish all you had planned, I love you anyway”) and gratitude for perceived positives (e.g., “I can choose my attitude … to be grateful for all I can do, there are always people hurting far worse than I”).

#### Struggling with Identity

Closely linked to the fighting and learning to live with FM themes was content detailing members’ struggle to find or regain a sense of self in the wake of FM. The struggling with identity theme prominently captured members’ descriptions of the person they were before FM, their struggle to continue living as that person, and their experience of grieving or letting go of their pre-FM identity. On rare occasions, members also described their experience reconceptualizing their identity as a person with FM.

Members often described themselves as active, health-conscious, hardworking, and productive people before FM (e.g., “I was a health nut, vegetarian, athletic and clean living until the fibro started”). These members concurrently expressed significant sadness and frustration at their perceived inability to embody those characteristics because of FM (e.g., “I miss the old me so much … realistically I’m never going to be 100% again and I’m never going to be the me I used to be”). Frustration, despair, and fear were expressed at the prospect of becoming their FM self forever, which members described as a flawed, shameful identity (e.g., “Living like this sucks and I hate it. I want the old ME back”; “I’m just tired of hurting, crying, missing people, missing out, feeling worthless, pretending I’m ok …”). For some, the emotional experience of this loss appeared to drive continued efforts to fight FM or self-identify as a “FM survivor” or “warrior.”

Others described adjusting their definitions of values like being fit and healthy so that it still resonated with their sense of self (e.g., “My new definition of TRUE HEALTH means … laughing [even at work], breathing, loving, and just living my LIFE without letting illness and stress break me down”). Some described a more gratitude- and present-focused approach to establishing a new sense of self. Exemplifying this latter point, one group member posted: “We need to take joy in how much knowledge we have gained and how much we have learned to appreciate what we still do have. I look forward to discovering the person I am yet to become.”

#### Struggling with Distressing Thoughts and Feelings

Members often discussed experiencing distressing thoughts and feelings about a myriad of topics. The nature and severity of these difficulties varied greatly and were not always explicitly linked to members’ FM symptoms or illness experience (e.g., some members discussed difficult family situations). Members often posted about emotional responses to everyday events in the context of FM (e.g., fear of medical procedures, frustration with mobility limitations). Others discussed more general struggles with negatively valanced thoughts (e.g., worthlessness), heightened worries or fears, or intense emotions that may constitute mental health symptoms. For example, one member posted: “… as soon as my head hits the pillow I remember and worry about everything I forgot to do … every hurtful thing that ever happened to me, every deed that I am sorry about. …” Another posted: “I’ve been not motivated to do anything, way quicker to anger … crying. … I haven’t cooked or cleaned anything … getting out of bed only when I have to go to work. …”

Oftentimes, members disclosed possible mental health symptoms or diagnoses in their list of health problems or as part of their question about whether these difficulties are normative for persons with FM. For example, one member asked, “Does anyone one else find their anxiety levels are much higher during a flare … finding my anxiety is more difficult to deal with then the pains at this point. …” Similarly, another member posted: “Does anyone find that after a bad pain spell the depression hits?” Responses to these posts generally included support, information, or advice on how to address emotional difficulties. Support and endorsement of passive coping strategies (e.g., resting, praying, rescheduling activities, and waiting for pain to pass) was frequently noted in the content extracted from all groups. For example, one user posted: “If you’re struggling you deserve to make self-care a priority. Whether that means lying in bed all day, eating comfort good, putting off homework, crying, sleeping, rescheduling plans … or doing nothing at all.”

Posts about mental health struggles often elicited discussion about the impact of mental health symptoms or stress on FM symptoms (e.g., “Thinking about things causes stress which causes pain …”). In some instances, content discussing past or present desire to die or escape life or previous suicide attempts was identified. Responses to this content generally urged the poster to seek professional help and offered support and encouragement.

References to mental health professionals (e.g., counselors, psychologists, psychiatrists) were rare and suggested that members may not fully understand the differences between various types of mental health services offered by various professionals (e.g., supportive counseling versus therapy for a mental health disorder). In one particularly salient discussion, group members suggested that consulting a mental health professional can be useful when attempting to make a disability insurance claim but indicated that counseling was generally unhelpful and that peers represent a superior option for mental health support (i.e., “[professional counselling is] not necessary, that’s what we’re here for”).

#### Judgment

Content describing experiences of judgment (i.e., being negatively evaluated or discriminated against due to FM-related stigmatization and stereotypes) was posted in all groups and generally elicited considerable discussion among group members. Posters often described the negative stereotypes they encountered about persons with pain or FM (e.g., lazy, malingerer, attention-seeking, crazy). They also described personal experiences of stigmatization and discrimination from spouses, family, friends, medical professionals, work colleagues/superiors, and insurance and government agencies. One member commented that “[her] neurologist doesn’t recognize FM … says it doesn’t exist” and another commented that she “had a doctor tell me it was all in my head years ago.” Another group members posted: “Family and friends don’t get it. I try not to say I don’t feel good or tired … I get told we are sick of hearing it.” When discussing stigma at work, a group member commented: “Some of us can work and some of us can’t. All of this does not make the person it doesn’t work for lazy, crazy or unwilling for change.” At times, members also disclosed negative judgments or stereotypes they had internalized (e.g., “I’m actually called Crazy [name], I just raise my hand and say YEP”).

Posts depicting judgment provoked strong emotional responses from group members as evidenced by the extent to which they generated comments (some posts received dozens of responses). Commenters typically offered support, advice, or descriptions of their own similar stigmatizing experiences (e.g., “I’m so tired of hearing how lazy I am … it breaks my heart, I used to work from sun up until sun down”; “When people think I am faking, I always wonder why anyone would want to fake something like this”).

Alternatively, some judgment posts were more general and declarative in nature (e.g., “Don’t always view someone’s complaints as a want for attention, sympathy, or validation. Sometimes someone’s complaints are just them longing desperately for a normal day with no pain and limitations”). These posts received fewer responses but generally were well “liked” (a Facebook feature that allows users to give a “thumbs up” response to posted content). Responses to judgment posts were generally supportive and almost always validating. Responses also occasionally conveyed gratitude for the existence of “our site,” which was described as a “safe place to go” to vent about experiences of judgment and to seek support from similarly affected others.

#### Empowerment-seeking

Members’ sought empowerment for themselves and others with FM via efforts to improve their understanding of FM (e.g., causes, treatments), be assertive, identify with positive attributes, and practice self-compassion. Two members of one group exemplified some of these points in their response to a peer struggling with family boundary-setting. They commented: “Setting boundaries with others is NOT your ‘ugly side’ … stand strong for yourself, you ROCK!” and “Be as committed and loving to yourself as you have been to those around you … it’s your life that you are fighting for. And you are worth it.”

Labeling persons with FM as strong and courageous (e.g., “people with chronic illness are freaking awesome and courageous,” “we are true warriors … I certainly admire our strength”) and practicing self- and other-validating skills were other forms of empowerment-seeking commonly described by group members. Posts demanding compassion from health providers/family/friends and respect for patient experiences and expertise were present but less common. Exemplifying these sentiments, one prominent poster wrote: “Do not let anybody try to tell you they know best or what you should be doing … they do not know what works best … you know your body better than anybody else.” This type of empowerment-seeking was often embedded in responses to peers’ descriptions of stigmatizing experiences with others. Stigmatizing encounters with medical professionals appeared to be particularly damaging to members’ willingness to strive for and place trust in patient–provider relationships, leading some members to describe the world outside their FM OPSG as hostile. In these cases, discussion typically shifted to advocacy (the final type of empowerment-seeking), with emphasis on uniting as a community to combat negative FM-related attitudes and discrimination. The following responses to one member’s post about a stigmatizing experience with a health provider exemplifies this shift:

Responder 1: United we stand … someday someone will listen and totally get it from our point of view.

Responder 2: These archaic uninformed “Drs” need to get with the program. Don’t put up with their smug BS. We know more about our condition than anyone else. We live it. We need to have our voice heard.

## Discussion

To the authors’ knowledge, the current study constitutes the first examination of an English-speaking Facebook FM OPSG despite the popularity of social media–based support seeking.^15^ An inductive analytic approach, which does not impose expectations or restrictions on to the analytic process, facilitated a deeper, multilayered picture of what group members discuss in FM OPSGs. Consistent with previous findings, the results of this study indicate that group members seek and offer FM-related emotional support and information (higher-order themes in the thematic hierarchy). In addition, the authors identified six lower-order themes describing particular issues FM OPSGs users seek information and support about: coping with judgment, identity, fighting FM, learning to live with FM, struggling with difficult thoughts and feeling, and empowerment-seeking. To the authors’ knowledge, no previous study had proposed a hierarchical framework describing the content of FM OPSG discussion, and no previous study has identified all of these lower-order themes. This study is also the first to directly compare discussion content from multiple FM OPSGs and to determine that the nature of discussions was thematically consistent across groups.

### Reasons Group Members Participate in Facebook FM OPSGs

Results from the present study identifying information and support-seeking as common discussion topics are consistent with previous studies of non-social media–based FM OPSGs^22,23^ and suggest that persons with FM turn to OPSGs to address unmet information and emotional support needs. This speaks to the resourcefulness and initiative of persons with FM while emphasizing the point that existing health resources may not be adequately meeting patients’ information and emotional support needs. The present findings further demonstrate that group members may at times be deferring to peers rather than professionals as primary sources of FM expertise and support. This is potentially problematic because, like other researchers,^33,34^ the authors found that the information posted in Facebook OPSGs was not consistently interpretable by persons without medical or other relevant education and did not consistently align with current evidence-based diagnostic and treatment approaches for FM. In peer-led groups, group leaders (i.e., admins or moderators) may not possess the knowledge, training, skills, and resources to moderate discussions to ensure that poor-quality information is not propagated.^16^ For these reasons, reliance on OPSGs as a primary source rather than as an adjuvant source of information about FM may carry unacknowledged risks.

In addition to information, group members typically seek and offer emotional support (e.g., empathy, compassion, connection, acceptance, encouragement, validation, belonging) in FM OPSGs. Validated emotional disclosures are associated with resilience to FM-related stigmatization and modest emotional (e.g., reduced depressive symptoms and interpersonal sensitivity), cognitive (e.g., perceived loneliness), and physiological benefits.^35–40^ The current findings suggest, however, that responses from group members may not always be perceived as validating, have lasting positive effects, or be universally helpful. For example, group members often responded to posts soliciting emotional support with unsolicited advice without concurrently conveying emotionally supportive sentiments. Given that persons with FM often feel stigmatized and invalidated by others’ coping suggestions,^41,42^ these types of responses may be perceived by the support-seeker as invalidating, unsupportive, and generally unhelpful.

Emotional disclosures or support-seeking in the form of venting, which was common in the FM OPSGs, may also be unhelpful or lead to responses perceived to be invalidating or unsupportive. Venting may occur commonly in FM OPSGs because offline audiences often respond intolerantly to pain-related venting.^41,43^ Following a distressing experience, venting to others is a relatively normal behavior motivated by a desire to receive emotional (e.g., validation, comfort, bonding, empathy) and cognitive support (e.g., help clarifying the meaning and significance of an experience, reframing/reappraising it, modifying associated goals and schemas).^44^ Responses to venting posts in the FM OPSGs examined herein conveyed emotional support associated with short-term emotional relief, but they rarely provided the cognitive processing supports provided in bona fide psychological interventions that are typically necessary for sustained emotional recovery.^37,44,45^ This is not surprising given that the provision of cognitive support typically requires professional training.

Moreover, frequent or collective venting among members may promote co-rumination, defined as “extensive and frequent discussion, speculation, and focus on negative feelings related to personal problems with a close friend.”^46(p133)^ Co-rumination can increase perceived friendship quality and satisfaction but can concurrently amplify anxious and depressive symptoms.^47,48^ This may be true even for members reading but not participating in venting discussions, resulting in an affective distress ripple effect within the group that extends beyond only those members posting content. Collectively, these findings suggest that FM OPSGs can provide much-needed emotional support and friendship to persons with FM but should not be considered an equivalent substitute for professional intervention, which generally addresses both emotional and cognitive support needs in a co-rumination free environment.^49^

### Reasons Group Members Seek Information or Support in a Facebook FM OPSGs

Although persons with FM have been seeking/offering information and support in FM OPSGs, little information exists describing the nature of group members’ informational and emotional support needs. That is, little is known about the issues or topics that underlie persons with FMs’ information and emotional support needs. This study addresses this gap in the literature by identifying content-focused themes that characterize FM OPSG users’ information and emotional support needs (i.e., fighting FM, learning to live with FM, struggling with identity, struggling with difficult thoughts and feelings, judgment, and empowerment-seeking).

The results indicate that many group members frequently sought/offered information and support to bolster their ability to fight or learn to live with FM. Engagement in discussions focused on fighting FM (i.e., resolving or minimizing symptoms of FM, especially pain) could promote activities that delay adjustment processes such as adaptive coping and acceptance.^50^ That is, the time and resources members expend discussing and experimenting with techniques to reduce symptoms (fighting FM) likely detracts in some ways from their capacity to invest in activities that give their life meaning and purpose and thus may be unintentionally harmful.

In contrast, learning to live with FM posts reflected members’ investment of time and resources in meaningful activities that foster improved quality of life and self-acceptance. These types of posts were far less frequent in this data set and have not been identified by previous researchers, perhaps because of methodological limitations. It is possible this theme has only recently arisen in FM OPSGs or is unique to Facebook FM OPSGs. In this case, these results may indicate that a small minority of persons with FM use contemporary OPSGs as a resource for promoting and supporting each other’s journeys toward illness acceptance.

Group members’ struggles with identity represent another novel finding regarding OPSG discussion content. Extant research indicates that persons with chronic pain often view pain as shameful, which hinders their ability to incorporate pain into a positive sense of self or identity.^51–55^ The prominence of this type of content in the OPSGs suggests that difficulties reconceptualizing a positive identity in the context of FM are common, distressing, and potentially an issue that is neglected by both researchers and support providers. The rare posts describing efforts to reconceptualize a positive identity that incorporates FM indicates that a select few members may be using the Facebook group as a source of information and support while navigating identity loss and reconceptualization.

FM-related shame and identity threat undoubtedly contribute to distressing thoughts and emotional upset.^51,55^ Chen^22^ and van Uden-Kraan and colleagues^23^ observed that members described struggling with difficult thoughts and feelings. The current, more nuanced findings, however, indicate there was considerable variability in the severity of distress group members described (e.g., mild anxiety and frustration versus despair and suicidal ideation). Posts depicting severe psychological symptoms may reflect contemporary society’s increased comfort with online communication and the degree of unmet mental health needs among FM populations. Group members experiencing emotional difficulties or psychological problems may also view their FM OPSG as the only accessible safe place to express their emotional distress and to seek advice and support.

The provision of emotional support (e.g., validation) without concurrent cognitive supports may make OPSGs appealing and beneficial in some ways because this type of support may be more palatable to persons who have been pervasively stigmatized and invalidated.^35,37,44^ For instance, group members are not required to participate in group discussions, adopt or practice skills, or make changes to palliate distress in an OPSG and can disengage (i.e., stop posting, delete their post, disengage from the group altogether, or delete their profile) if they feel pressured to do so.^56^ Thus, this “support without pressure to take action” environment may attract persons struggling with emotional difficulties who are in the early stages of change (i.e., precontemplation, contemplation, and determination) and in need of emotional support as a preparatory step toward being ready to engage with cognitive supports.^57,58^

This environment may concurrently and unintentionally facilitate disclosure of symptoms like suicidal thoughts and despair even though timely, supportive responses are not guaranteed. This is problematic because OPSG members are not typically trained in mental health intervention and are unlikely to possess the physical and emotional resources to respond to such disclosures.^56^ Expressions of intense emotional distress without opportunities for upward social comparison can lead to increased hopelessness and emotional distress and a focus on symptoms within OPSGs.^34,59^ Thus, it is likely that these posts do not adequately address the needs of the poster and may unintentionally produce stress, sadness, helplessness, and worry in readers who joined the FM OPSG primarily to seek support themselves.^34,59,60^

Many of the distressing thoughts and feelings members described were connected to experiences of FM-related stigmatization and discrimination, which underscored the theme judgment. These experiences were a frequent topic of support-seeking (especially venting) posts, which suggests that members may find judgment especially difficult to cope with. Frequent and chronic exposure to judgment may cause some members to internalize stigmatizing beliefs about themselves and FM and withdraw from social contact, inadvertently creating the ideal conditions for depressive symptoms to flourish. Thus, the identification of more severe psychological symptoms in these Facebook OPSGs may reflect the mental health consequences of chronic exposure to FM-related stigmatization and discrimination combined with a lack of accessible, available resources to help persons with FM build and maintain their psychological resiliency. These findings emphasize the importance of reducing FM-related stigma and discrimination and highlight judgment as an important yet rarely addressed discussion topic for many persons with FM.

Like Barker,^24^ the authors noted that FM group members often discussed obstacles (including judgment) they encountered while striving to (re)gain a sense of empowerment. Generally, empowerment in the context of illness and disability requires role clarity in patient–health provider relationships, patients’ capacity to understand their condition and engage in a collaborative health relationship, and a supportive environment.^61^ FM’s unclear etiology and stigmatized status may render these empowerment milestones out of reach for many FM OPSG members.^62^ Instead, these findings suggest that FM OPSGs provide a setting where members can begin rebuilding a sense of empowerment within the constraints imposed by their stigmatized illness. Using an FM OPSG as a platform to advocate for compassion/respect may help to validate and normalize others’ “experiences of disempowerment” and fuel adaptive empowerment-seeking action.^63,64^

In some cases, however, advocacy posts conveyed (intentionally or not) an us-versus-them mentality. This is potentially problematic because this stance may negatively bias members’ capacities to reframe their own or others’ negative interpersonal experiences, prompting members to view individuals without FM (e.g., physicians, employers) as a threatening outgroup.^24,65,66^ FM OPSGs may unintentionally foster this type of mentality because they permit members to commiserate about negative interpersonal experiences, effectively creating a digital anthology of perceived mistreatment by outgroup members. Participation in the group may therefore instill or strengthen negative views about persons outside the OPSG (including helping professionals).

### Limitations and Future Directions

A consequence of using a noninteractive, observational approach is an inability to collect demographic information (including confirmation of FM diagnosis) that would help to further contextualize these findings and the inability to test whether the OPSG discussions produce adaptive or maladaptive coping in members’ offline lives. Likewise, the authors’ analyses did not incorporate a process viewpoint; the authors focused on understanding what was discussed overall and did not examine the interpersonal nuances of members reactions to one another. Follow-up research could help to determine how the support and other benefits offered by the group as well as the costs of participation (e.g., exposure to venting, others’ experiences of judgment) affect group members’ offline behaviors. Follow-up research may also help to identify additional costs and benefits of participation that are undetectable by reviewing discussion content alone.

Exploration of groups with a specific coping stance or religious affiliation should also be explored because the content and potential costs and benefits of participation in these types of OPSGs may differ significantly from the groups described here. Interactive research (e.g., interview studies) could also improve the current understanding of how FM OPSG participation influences (or does not influence) members’ offline illness experiences, which was beyond the scope of this study. In addition, follow-up interactive research soliciting a patient perspective on these findings, which were interpreted through the lenses of two female mental health care providers, could provide a more complete picture of the nature of discussions in Facebook FM OPSGs. This research could also help to identify discrepancies between patient and provider community perspectives that may have implications for patient–provider relationships and patient-centered care delivery.

Additional follow-up research may be especially important for establishing how men, who are less frequently diagnosed with FM, experience FM OPSGs. The majority of content examined in this research was posted by individuals who appeared to be women (based on their picture, name, or information they disclosed). It is very possible that men’s experiences of FM OPSGs differ from women’s experiences and that the discussion content of a group established for men with FM would differ from that of the groups reviewed here. Finally, discussion content in other FM OPSGs on Facebook or other online platforms may differ significantly. Without additional research, assumed transferability of these findings is discouraged.

### Conclusions

The findings herein suggest that participation in an FM OPSG has potential benefits and pitfalls. From a positive standpoint, FM OPSGs appear to provide members with opportunities to receive much-needed emotional support (e.g., validation, acceptance, connection), which may be lacking in many members’ offline lives. These findings also suggest that the FM OPSGs provide emotional support that is easily digested by persons who have been pervasively stigmatized or invalidated but do not provide cognitive supports that are typically needed for sustained emotional recovery. The groups simultaneously provide members the chance to offer support and information (including experiential knowledge and empowerment strategies) to others, which may allow members to feel empowered and useful and help them to find meaning in their FM experience. Sporadically, members also shared their experience of learning to live with FM and formulating a positive with-FM identity, which may encourage other members to adopt practices that align more closely with chronic pain acceptance. On the other hand, the findings also suggest members of an FM OPSG may be exposed to content that is misleading or inaccurate (i.e., regarding the causes and treatment of FM) or that generates or intensifies distressing thoughts and feelings (e.g., posts depicting experiences of judgment, suicidal ideation, or anger). Discussion content in the FM OPSGs may also promote more negative attitudes toward people without FM and support maladaptive illness management strategies (e.g., “fighting” adjustment to FM).

These findings may be useful to patients considering the potential costs and benefits of joining an FM OPSG and to health providers helping patients find online resources that can address their emotional or psychological support needs. Providers are cautioned against assuming that all FM OPSGs are inherently harmful or beneficial to any patient’s well-being. Providers advising patients navigating OPSGs are encouraged to emphasize that, ideally, peer support environments provide role models who function as psychologically stable sources of upward comparison that help instill hope and empowerment to facilitate positive change in those seeking support.^67^ Without these role models to act as support providers, support communities are more vulnerable to being less helpful or even distressing to members.^34,59^

These findings also point to several possible areas for targeted services such as improving patients’ capacities to evaluate and identify evidence-based online information shared about FM (see evaluation tool for patients suggested by Bailey and colleagues^68^) and psychoeducation for family members and health providers on FM and FM-related stigmatization. These findings also emphasize the importance of providing explicit emotional support (e.g., validation) and space to discuss important issues (e.g., FM’s impact on identity, finding empowerment in the context of FM) when supporting members of this clinical population both professionally and informally. Of note, it is evident persons with FM are seeking information and emotional support in relation to serious mental health symptoms (e.g., suicidal ideation, despair) from online peers who are likely ill-equipped to provide the level of support and intervention needed for recovery. This implies an acute need for access to appropriate mental health services for this population. Given that persons with FM are already accessing online peer supports and OPSGs are an accessible and potential cost-effective resource for patients, further investigation of FM OPSGs and their potential clinical utility could be very fruitful.

## References

[1] Bazzichi L, Giacomelli C, Consensi A, Giorgi V, Batticciotto A, Di Franco M, Sarzi-Puttini P. One year in review 2020: fibromyalgia. Clin Exp Rheumatol. 2020;38:S3–8. 32116216

[2] Choy E, Perrot S, Leon T, Kaplan J, Petersel D, Ginovker A, Kramer E. A patient survey of the impact of fibromyalgia and the journey to diagnosis. BMC Health Serv Res. 2010;10(102):1–9. doi:10.1186/1472-6963-10-102. 20420681 PMC2874550

[3] Arnold LM, Crofford LJ, Mease PJ, Burgess SM, Palmer SC, Abetz L, Martin SA. Patient perspectives on the impact of fibromyalgia. Patient Educ Couns. 2008;73(1):114–20. doi:10.1016/j.pec.2008.06.005. 18640807 PMC2564867

[4] Sim J, Madden S. Illness experience in fibromyalgia syndrome: a metasynthesis of qualitative studies. Soc Sci Med. 2008;67(1):57–67. doi:10.1016/j.socscimed.2008.03.003. 18423826

[5] Asbring P, Närvänen AL. Women’s experiences of stigma in relation to chronic fatigue syndrome and fibromyalgia. Qual Health Res. 2002;12(2):148–60. doi:10.1177/104973230201200202. 11837367

[6] Kool MB, Geenen R. Loneliness in patients with rheumatic diseases: the significance of invalidation and lack of social support. J Psychol. 2012;146(1–2):229–41. doi:10.1080/00223980.2011.606434. 22303622

[7] Agarwal A, Oparin Y, Glick L, Fitzcharles MA, Adachi JD, Cooper MD, Gallo L, Wong L, Busse JW. Attitudes toward and management of fibromyalgia: a national survey of Canadian rheumatologists and critical appraisal of guidelines. J Clin Rheumatol. 2018;24(5):243–49. doi:10.1097/RHU.0000000000000679. 29280818

[8] Arnold LM, Choy E, Clauw DJ, Oka H, Whalen E, Semel D, Pauer L, Knapp L. An evidence-based review of pregabalin for the treatment of fibromyalgia. Curr Med Res Opin. 2018;34(8):1397–409. doi:10.1080/03007995.2018.1450743. 29519159

[9] Welsch P, Üçeyler N, Klose P, Walitt B, Häuser W. Serotonin and noradrenaline reuptake inhibitors (SNRIs) for fibromyalgia. Cochrane Database Syst Rev. 2018;2:CD010292. doi:10.1002/14651858.CD010292.pub2. 29489029 PMC5846183

[10] Fitzcharles MA, Ste-Marie PA, Goldenberg DL, Pereira JX, Abbey S, Choinière M, Ko G, Mouin DE, Panopalis P, Proulx J, et al. the National Fibromyalgia Guideline Advisory Panel. 2012 Canadian guidelines for the diagnosis and management of fibromyalgia syndrome: executive summary. Pain Res Manag. 2013;18(3):119–26. doi:10.1155/2013/918216. 23748251 PMC3673928

[11] Hann KEJ, McCracken LM. A systematic review of randomized controlled trials of acceptance and commitment therapy for adults with chronic pain: outcome domains, design quality, and efficacy. J Contextual Behav Sci. 2014;3(4):217–27. doi:10.1016/j.jcbs.2014.10.001.

[12] Bernardy K, Klose P, Welsch P, Häuser W. Efficacy, acceptability and safety of cognitive behavioral therapies in fibromyalgia syndrome – a systematic review and meta-analysis of randomized controlled trials. Eur J Pain. 2018;22(2):242–60. doi:10.1002/ejp.1121. 28984402

[13] Glombiewski JA, Sawyer AT, Gutermann J, Koenig K, Rief W, Hofmann SG. Psychological treatments for fibromyalgia: a meta-analysis. PAIN. 2010;151(2):280–95. doi:10.1016/j.pain.2010.06.011. 20727679

[14] Cudney SA, Butler MR, Weinert C, Sullivan T. Ten rural women living with fibromyalgia tell it like it is. Holist Nurs Pract. 2002;16(3):35–45. doi:10.1097/00004650-200204000-00009. 11913226

[15] Berard AA, Smith AP. Post your journey: instagram as a support community for people with fibromyalgia. Qual Health Res. 2019;29(2):237–47. doi:10.1177/1049732318789102. 30066603

[16] Gupta T, Schapira L. Online communities as sources of peer support for people living with cancer: a commentary. J Oncol Pract. 2018;14(12):725–30. doi:10.1200/JOP.18.00261. 30335558

[17] Chou WYS, Gaysynsky A, Trivedi N, Vanderpool RC. Using social media for health: national data from HINTS 2019. J Health Commun. 2021;26(3):184–93. doi:10.1080/10810730.2021.1903627. 33856286 PMC12645447

[18] Meta. How do I join a Facebook group as my profile or my page? Meta; 2021[accessed 2021 Nov 24]. https://www.facebook.com/help/103763583048280

[19] Meta. How do I choose what I’m notified about in a Facebook group? Meta; 2021[accessed 2021 Nov 24]. https://www.facebook.com/help/187225274663021/?helpref=search

[20] Ridings C, Gefen D, Arinze B. Psychological barriers: lurker and poster motivation and behavior in online communities. Commun Assoc Inf System. 2006;18(16):329–54. doi:10.17705/1CAIS.01816.

[21] Moretti FA, Silva SS, Novoa CG. Characteristics and perception of social support by patients with fibromyalgia in Facebook. Br J Pain. 2018;1:4–8.

[22] Chen AT. Exploring online support spaces: using cluster analysis to examine breast cancer, diabetes, and fibromyalgia support groups. Patient Educ Couns. 2012;87(2):250–57. doi:10.1016/j.pec.2011.08.017. 21930359

[23] van Uden-Kraan CF, Drossaert CHC, Taal E, Lebrun CEI, Drossaers-Bakker KW, Smit WM, Seydel ER, van de Laar MAFJ. Coping with somatic illnesses in online support groups: do the feared disadvantages actually occur? Comput Hum Behav. 2008;24(2):309–24. doi:10.1016/j.chb.2007.01.014.

[24] Barker KK. Electronic support groups, patient-consumers, and medicalization: the case of contested illness. J Health Soc Behav. 2008;49(1):20–36. doi:10.1177/002214650804900103. 18418983

[25] Barak A, Grohol JM. Methodology, validity, and applicability: a critique on Eysenbach. Qual Saf Health Care. 2004;328:1166. doi:10.1136/bmj.328.7449.1166.

[26] Canadian Institutes of Health Research, Natural Sciences and Engineering Research Council of Canada, and Social Sciences and Humanities Research Council. Tri-Council policy statement: ethical conduct for research involving humans. Ottawa: Secretariat on Responsible Conduct of Research, Government of Canada; 2018 Dec [accessed 2021 Jul 3]. https://ethics.gc.ca/eng/policy-politique_tcps2-eptc2_2018.html

[27] Eysenbach G, Till JE. Ethical issues in qualitative research on internet communities. BMJ. 2001;323(7321):1103–05. doi:10.1136/bmj.323.7321.1103. 11701577 PMC59687

[28] British Psychological Society. Code of human research ethics. London(UK): British Psychological Society; 2021 Apr [accessed 2021 Jul 3]. https://www.bps.org.uk/sites/bps.org.uk/files/Policy/Policy%20-%20Files/BPS%20Code%20of%20Human%20Research%20Ethics.pdf

[29] Braun V, Clarke V. Using thematic analysis in psychology. Qual Res Psychol. 2006;3(2):77–101. doi:10.1191/1478088706qp063oa.

[30] Korstjens I, Moser A. Series: practical guidance to qualitative research. Part 4: trustworthiness and publishing. Eur J Gen Pract. 2018;24(1):120–24. doi:10.1080/13814788.2017.1375092. 29202616 PMC8816392

[31] Lincoln YS, Guba EG. Establishing trustworthiness. In: Lincoln YS, Guba EG, editors. Naturalistic inquiry. Beverly Hills (CA): Sage; 1985. p. 289–331.

[32] Stiles B. Quality control in qualitative research. Clin Psychol Rev. 1993;13(6):593–618. doi:10.1016/0272-7358(93)90048-Q.

[33] Naslund JA, Bondre A, Torous J, Aschbrenner KA. Social media and mental health: benefits, risks, and opportunities for research and practice. J Technol Behav Sci. 2020;5(3):245–57. doi:10.1007/s41347-020-00134-x. 33415185 PMC7785056

[34] Tan YT, Rehm IC, Stevenson JL, De Foe A. Social media peer support groups for obsessive-compulsive and related disorders: understanding the predictors of negative experiences. J Affect Disord. 2021;281:661–72. doi:10.1016/j.jad.2020.11.094. 33234279

[35] Lepore SJ, Fernandez-Berrocal P, Ragan J, Ramos N. It’s not that bad: social challenges to emotional disclosure enhance adjustment to stress. Anxiety Stress Coping. 2004;17(4):341–61. doi:10.1080/10615800412331318625.

[36] Lepore SJ, Ragan JD, Jones S. Talking facilitates cognitive–emotional processes of adaptation to an acute stressor. J Pers Soc Psychol. 2000;78(3):499–508. doi:10.1037/0022-3514.78.3.499. 10743876

[37] Nils F, Rimé B. Beyond the myth of venting: social sharing modes determine the benefits of emotional disclosure. Eur J Soc. 2012;42(6):672–81. doi:10.1002/ejsp.1880.

[38] Ochocka J, Nelson G, Janzen R, Trainor J. A longitudinal study of mental health consumer/survivor initiatives: part 3 – a qualitative study of impacts of participation on new members. J Community Psychol. 2006;34(3):273–83. doi:10.1002/jcop.20099.

[39] Radcliffe AM, Lumley MA, Kendall J, Stevenson JK, Beltran J. Written emotional disclosure: testing whether social disclosure matters. J Soc Clin Psychol. 2007;26(3):362–84. doi:10.1521/jscp.2007.26.3.362. PMC2932452 20824150

[40] Repper J, Carter T. A review of the literature on peer support in mental health services. J Ment Health. 2011;20(4):392–411. doi:10.3109/09638237.2011.583947. 21770786

[41] Armentor JL. Living with a contested, stigmatized illness: experiences of managing relationships among women with fibromyalgia. Qual Health Res. 2017;27(4):462–73. doi:10.1177/1049732315620160. 26667880

[42] Kool MB, van Middendorp H, Boeije HR, Geenen R. Understanding the lack of understanding: invalidation from the perspective of the patient with fibromyalgia. Arthritic Care Res. 2009;61(12):1650–56. doi:10.1002/art.24922. 19950317

[43] Herbette G, Rimé B. Verbalization of emotion in chronic pain patients and their psychological adjustment. J Health Psychol. 2004;9(5):661–76. doi:10.1177/1359105304045378. 15310420

[44] Rimé B. Emotion elicits the social sharing of emotion: theory and empirical review. Emot Rev. 2009;1(1):60–85. doi:10.1177/1754073908097189.

[45] Kennedy-Moore E, Watson JC. How and when does emotional expression help? Rev Gen Psychol. 2001;5(3):187–212. doi:10.1037/1089-2680.5.3.187.

[46] Carlucci L, D’Ambrosio I, Innamorati M, Saggino A, Balsamo M. Co-rumination, anxiety, and maladaptive cognitive schemas: when friendship can hurt. Psychol Res Behav Manag. 2018;11:133–44. doi:10.2147/PRBM.S144907. 29692638 PMC5903493

[47] Calmes CA, Roberts JE. Rumination in interpersonal relationships: does co-rumination explain gender differences in emotional distress and relationship satisfaction among college students? Cognit Ther Res. 2008;32:577–90. doi:10.1007/s10608-008-9200-3.

[48] Haggard DL, Robert C, Rose AJ. Co-rumination in the workplace: adjustment trade-offs for men and women who engage in excessive discussions of workplace problems. J Bus Psychol. 2011;26(1):27–40. doi:10.1007/s10869-010-9169-2.

[49] Antony MM, Barlow DH, Tung ES, Wang M, Brown TA, Rellini AJ, editors. Transdiagnostic assessment of emotional. Handbook of assessment and treatment planning for psychological disorders. 3rd ed. New York (NY): Guilford; 2020. 119–137.

[50] Hayes SC, Luoma JB, Bond FW, Masuda A, Lillis J. Acceptance and commitment therapy: model, processes and outcomes. Behav Res Ther. 2006;44(1):1–25. doi:10.1016/j.brat.2005.06.006. 16300724

[51] Crump L, LaChapelle DL. Incorporating pain experiences into personal identity: implications for romantic relationships and securing social support. Poster presented at: 38th Annual Scientific Meeting of the Canadian Pain Society; 2017 May 23-26; Halifax (NS).

[52] Ferguson T, Dempsey H, Ashbaker M. Unwanted identities: a key variable in shame–anger links and gender differences in shame. Sex Roles. 2000;42:133–57. doi:10.1023/A:1007061505251.

[53] Risdon A, Eccleston C, Crombez G, McCracken L. How can we learn to live with pain? A Q-methodological analysis of the diverse understandings of acceptance of chronic pain. Soc Sci Med. 2003;56(2):375–86. doi:10.1016/S0277-9536(02)00043-6. 12473322

[54] Richardson JC. Establishing the (extra)ordinary in chronic widespread pain. Health. 2005;9(1):31–48. doi:10.1177/1363459305048096. 15576423

[55] Smith JA, Osborn M. Pain as an assault on the self: an interpretative phenomenological analysis of the psychological impact of chronic benign low back pain. Psychol Health. 2007;22(5):517–34. doi:10.1080/14768320600941756.

[56] Ma X, Hancock J, Naaman M. Anonymity, intimacy and self-disclosure in social media. Proceedings of 2016 CHI Conference on Human Factors in Computing Systems 2016; 2016 May 7-12; San Jose (CA). New York (NY): Association for Computing Machinery, p. 3857–69.

[57] Prochaska JO, Redding CA, Evers KE. The transtheoretical model and stages of change. In: Glanz K, Romer BK, Wiswanath K editors. Health behavior: theory, research, and practice. 4th ed. San Francisco (CA): John Wiley & Sons; 2015. p. 97–121.

[58] Prochaska JO, Velicer WF, Rossi JS, Goldstein MG, Marcus BH, Rakowski W, Fiore C, Harlow LL, Redding CA, Rosenbloom D, et al. Stages of change and decisional balance for 12 problem behaviors. Health Psychol. 1994;13(1):39–46. doi:10.1037/0278-6133.13.1.39. 8168470

[59] Salzer MS, Palmer SC, Kaplan K, Brusilovskiy E, Ten Have T, Hampshire M, Metz J, Coyne JC. A randomized, controlled study of Internet peer-to-peer interactions among women newly diagnosed with breast cancer. Psychooncology. 2010;19(4):441–46. doi:10.1002/pon.1586. 19484712

[60] Naslund JA, Aschbrenner KA, Marsch LA, Bartels SJ. The future of mental health care: peer-to-peer support and social media. Epidemiol Psychiatr Sci. 2016;25(2):113–22. doi:10.1017/S2045796015001067. 26744309 PMC4830464

[61] World Health Organization. WHO guidelines on hand hygiene in health care. Geneva (Switzerland): World Health Organization; 2009 [accessed 2021 Jul 2]. https://www.who.int/publications/i/item/9789241597906

[62] Chiauzzi E, DasMahapatra P, Cochin E, Bunce M, Khoury R, Dave P. Factors in patient empowerment: a survey of an online patient research network. Patient. 2016;9(6):511–23. doi:10.1007/s40271-016-0171-2. 27155887 PMC5107186

[63] Ball TC, Nario-Redmond MR. Positive social identity interventions: finding a conduit for well-being in stigmatized group memberships. In: Parks AC, Schueller SM, editors. The Wiley Blackwell handbook of positive psychological interventions. Chichester (UK): John Wiley & Sons; 2014. p. 327–43.

[64] Latrofa M, Vaes J, Pastore M, Cadinu M. “United we stand, divided we fall”! The protective function of self-stereotyping for stigmatised members’ psychological well-being. Appl Psychol. 2009;58:84–104. doi:10.1111/j.1464-0597.2008.00383.x.

[65] Cruwys T, Gunaseelan S. “Depression is who I am”: mental illness identity, stigma and wellbeing. J Affect Disord. 2016;189:36–42. doi:10.1016/j.jad.2015.09.012. 26402345

[66] Latrofa M, Vaes J, Cadinu MS-S. The central role of an ingroup threatening identity. J Soc Psychol. 2012;152:92–111. doi:10.1080/00224545.2011.565382. 22308763

[67] Soloman P. Peer support/peer provided services underlying processes, benefits, and critical ingredients. Psychiatr Rehabil J. 2004;27(4):392–401. doi:10.1002/pon.1586. 15222150

[68] Bailey SJ, LaChapelle DL, LeFort SM, Gordon A, Hadjistavropoulos T. Evaluation of chronic pain-related information available to consumers on the internet. Pain Med. 2013;14:855–64. doi:10.1111/pme.12087. 23565667

[69] NVivo qualitative data analysis software. 12 ed: QSR International Pty Ltd. 2018.

